# Psychological hardiness among deaf and hard-of-hearing female students in Saudi Arabia: a mixed-methods analysis of influencing factors and enhancement strategies

**DOI:** 10.3389/fpsyg.2026.1831967

**Published:** 2026-05-26

**Authors:** Nourah Ibrahim Albash, Reem Abdullatif Alarfaj

**Affiliations:** 1 Special Education Department, College of Education, King Faisal University, Al-Ahsa, Saudi Arabia; 2 Department of Education and Psychology, College of Education, King Faisal University, Al-Ahsa, Saudi Arabia

**Keywords:** deaf and hard-of-hearing, hearing impairment, mixed-methods, psychological hardiness, Saudi Arabia

## Abstract

Psychological hardiness is considered a critical psychological construct that supports the ability of deaf and hard-of-hearing (DHH) female students to adapt to personal, social, and educational challenges. This study aimed to determine the level of psychological hardiness, examine the factors influencing it, and explore the strategies used to enhance it among DHH female students in the Kingdom of Saudi Arabia. An explanatory sequential design, one of the mixed-methods research designs, was employed in this study. Quantitative data were collected using a scale developed by the researchers and administered to 69 DHH female students to assess their levels of psychological hardiness based on their responses. Subsequently, qualitative data were collected through in-depth individual interviews with 25 participants who had completed the scale. The interview questions were designed to obtain deeper insights from this purposively selected group regarding the factors influencing psychological hardiness and the strategies used by the students to strengthen it. The quantitative findings guided the selection of the qualitative sample and supported the interpretation of the qualitative analysis results. Quantitative data were analyzed using frequencies, means, and standard deviations to determine the level of psychological hardiness. The qualitative data were analyzed using thematic analysis. The quantitative findings revealed that the overall level of psychological hardiness among DHH female students was moderate, with a mean score of 2.87. The qualitative findings identified four key factors influencing psychological hardiness: family factors, societal factors, peer relationships, and teachers. The results further indicated that students employ several strategies to enhance psychological hardiness, including faith in God and reliance on Him, social support and relationship strengthening, coping and stress-management strategies, self-confidence and emotional regulation, and self-development and personal growth. These findings highlight the importance of developing supportive psychological and educational interventions that consider contextual factors and leverage effective coping strategies among this population.

## 1 Introduction

The contemporary world is witnessing rapid social and technological transformations, which have generated numerous challenges and pressures that affect individuals’ lives and their ability to adapt to increasing life demands. These changes have been associated with rising psychological stress making the maintenance of psychological balance and mental wellbeing increasingly complex (Ghoneim, 2015). During times of crisis and exposure to stressful situations, individuals may experience psychological disturbances that can undermine their emotional stability and adversely affect their productivity. At the same time, such pressures cannot be completely avoided, as withdrawing from them may reduce an individual’s effectiveness and overall functioning (Al-Hadi, 2020).

In addition to the general pressures experienced by individuals in society, deaf and hard-of-hearing (DHH) individuals face additional challenges related to the nature of their hearing impairment, including communication barriers, limited access to services, and experiences of social marginalization or discrimination. These factors may negatively affect their mental health and their ability to cope with everyday life demands (Crowe, 2022). Several studies indicate that hearing loss is associated with an increased risk of depression and cognitive decline. Moreover, social isolation and limited participation in social activities represent key pathways explaining the relationship between hearing loss and declining mental health (Fulton et al., 2015; Livingston et al., 2020; West, 2017).

In addition, some DHH individuals may experience traumatic events and perceived social discrimination, which may negatively affect self-esteem and increase the likelihood of social withdrawal or avoidance of social interaction (Anderson et al., 2018; Johnson et al., 2018; Mousley and Chaudoir, 2018; Shakarchi et al., 2020). Research evidence also suggests that DHH individuals tend to report higher levels of anxiety, depression, and anger, along with lower levels of life satisfaction compared with their hearing peers, which may affect their psychological and social adjustment (Al-Mukhambetova et al., 2021; Tsou et al., 2021).

Some of these challenges are also associated with language development limitations among certain DHH individuals. Language serves as a fundamental tool for social interaction and the acquisition of experiences, and any limitations in language development may restrict opportunities for effective communication with the surrounding social environment (Brice et al., 2011; Crowe, 2019; Hall et al., 2017). Furthermore, language deprivation during early childhood may affect cognitive and emotional development, thereby limiting the development of coping skills and psychological resilience among DHH individuals (Johnson et al., 2018). Additionally, certain social practices, such as audism or excessive preference for spoken language, may increase psychological stress among this population (Crowe, 2019; Mousley and Chaudoir, 2018). Similarly, Hard-of-hearing (HOH) individuals may experience difficulties in interpreting environmental stimuli and responding appropriately due to language limitations, which may lead to emotional instability or increased negative emotional experiences (Badr Al-Din et al., 2022).

Within this context, psychological hardiness represents an important psychological resource that helps individuals cope with stress and adapt effectively to crises. It reflects an individual’s ability to respond positively to difficult situations through the components of commitment, control, and challenge (Al-Hadi, 2020; Ghoneim, 2015; Mokhaimer, 2011). Psychological hardiness also mitigates the negative effects of stressful or traumatic events and enhances individuals’ ability to restore psychological balance and maintain mental wellbeing (Iacoviello and Charney, 2020; McKenzie and Reed, 2017).

Psychological hardiness is particularly important for DHH individuals, as it is influenced by several psychological and social factors, including problem-solving skills, emotional regulation, supportive social relationships, the availability of economic resources, and positive beliefs that help individuals find meaning in challenging experiences (Brice et al., 2011; Crowe, 2019; Johnson et al., 2018). Moreover, personal characteristics such as independence, motivation, and positive identity contribute to strengthening psychological hardiness, while belonging to a supportive community enhances psychological wellbeing (Ma et al., 2022; Sheridan, 2001). Access to appropriate information and services may also empower DHH individuals to develop effective coping strategies that enhance their psychological hardiness (Johnson et al., 2018).

Accordingly, the present study aims to identify the level of psychological hardiness among DHH female students, examine the factors influencing its development, and explore the strategies that may contribute to strengthening it, thereby supporting their ability to cope with life stressors and achieve better psychological wellbeing and personal adjustment.

## 2 Research problem

Deaf and hard-of-hearing individuals are more vulnerable to psychological and emotional difficulties, such as psychological distress, emotional instability, and social anxiety, compared with their hearing peers (Batten et al., 2014; Cheng et al., 2019; Fellinger et al., 2012). Some may also experience lower levels of self-esteem, self-efficacy, and perceived personal control, which may lead to social withdrawal and reduced social participation (Gao et al., 2023).

In addressing these challenges, psychological hardiness emerges as an important positive psychological construct in the field of mental health, as it helps individuals regulate negative emotions, adapt to stressful situations, and develop effective strategies for coping with future stressors (Fletcher and Sarkar, 2013). Despite the significance of this topic, the literature indicates a limited number of studies addressing psychological hardiness, resilience, and subjective wellbeing among this population (Crowe, 2022; Gao et al., 2020; West, 2017; Xu et al., 2025). Moreover, studies examining the relationship between hearing loss and psychological hardiness remain limited and present inconsistent findings, and this relationship has not been thoroughly explored, particularly regarding the potential role of psychological hardiness as a mediating or moderating variable in the relationship between hearing loss and mental health or subjective wellbeing (Bott and Saunders, 2021; Gao et al., 2023; Shukla et al., 2020).

Although some studies have investigated psychological hardiness among DHH individuals and its relationship with various psychological and social variables (Abdulrahman et al., 2021; Bazah et al., 2023; Crowe, 2022; Gao et al., 2023; Mousa and Khalaf, 2017), they have not directly examined the factors and strategies influencing the development of psychological hardiness among HOH female students in particular -According to the researchers’ knowledge-. Additionally, (Xu et al., 2025) highlighted the continued lack of understanding regarding the role of supportive variables, such as social support, in enhancing psychological resilience among this population.

Therefore, there is a need to investigate the level of psychological hardiness among DHH female students and to analyze the factors associated with it, as well as the strategies that may contribute to strengthening it. To address these gaps, the present study adopts an explanatory sequential mixed-methods design that integrates quantitative and qualitative approaches. While quantitative data are used to determine the level of psychological hardiness, the qualitative component explores contextual factors and strategies used to enhance it. This integrative approach allows for a more comprehensive understanding of psychological hardiness as both a measurable psychological construct and a lived experience embedded within a specific social context.

This study contributes theoretically to the literature on psychological hardiness among HOH female students by examining the interaction between psychological hardiness and related psychological factors. Practically, the findings aim to assist educators and policymakers in adopting the proposed adaptive strategies as a framework for enhancing psychological hardiness among these students, thereby improving their mental wellbeing and quality of life within Saudi schools.

### 2.1 Research questions

#### 2.1.1 Quantitative research questions

1. What is the level of psychological hardiness among DHH female students in Saudi Arabia?

#### 2.1.2 Qualitative research questions

1. What factors influence psychological hardiness among HOH female students in Saudi Arabia?

2. What strategies contribute to enhancing psychological hardiness among HOH female students in Saudi Arabia?

#### 2.1.3 Mixed-methods integration question

1. How do the influencing factors and supportive strategies contribute to explaining the level of psychological hardiness among HOH female students in Saudi Arabia?

## 3 Methodology

### 3.1 Research design

The present study adopted a sequential explanatory mixed-methods design to achieve a more comprehensive and in-depth understanding of the phenomenon under investigation. This design is widely used in educational, psychological, and social research, as it enables the integration of quantitative and qualitative data and allows researchers to capitalize on the strengths of both approaches in order to interpret the research phenomenon more accurately (Creswell, 2015). This design consists of two main phases. The study begins with a quantitative phase, which aims to collect initial data about the phenomenon under study, followed by a qualitative phase, which seeks to provide a deeper interpretation of the quantitative findings by exploring participants’ experiences and perspectives. The two phases are connected through the use of quantitative findings to guide participant selection for the qualitative phase and to inform the development of qualitative data collection tools (Creswell, 2015).

Methodological literature indicates that mixed-methods research designs are typically built around three major dimensions: sequence, priority, and integration. The dimension of sequence determines the order in which quantitative and qualitative data are collected, whereas priority refers to the relative emphasis assigned to each strand in addressing the research questions. Integration, in turn, concerns how and at what stage quantitative and qualitative data are brought together throughout the research process (Creswell, 2015). Given the nature of the research questions and the objectives of the study, the sequential explanatory design was considered the most appropriate approach. Quantitative data were collected first to provide an overall picture of the level of psychological hardiness among DHH female students. These findings also informed the selection of participants for the qualitative phase and the development of the interview questions (Creswell and Plano Clark, 2011). Because understanding the factors influencing psychological hardiness and the strategies used to enhance it required deeper exploration of participants’ lived experiences, the qualitative phase was given interpretive priority. In the final stage, the quantitative and qualitative findings were integrated during interpretation to generate a more comprehensive understanding of the phenomenon under study.

Accordingly, the study proceeded in two main phases: Quantitative phase: to identify the level of psychological hardiness among DHH female students. Qualitative phase: to explore the factors influencing psychological hardiness and the strategies used to enhance it, based on the quantitative findings.

### 3.2 Participants and sampling

A census sampling approach was used to collect data from the study population, which included all DHH female students enrolled in inclusive middle and secondary schools in Al-Ahsa Governorate, Saudi Arabia, during the 2025–2026 academic year. According to official statistics issued by the Ministry of Education, the total number of students in this population was 74. Five students were excluded, resulting in a final quantitative sample of 69 students, representing approximately 93% of the target population. This high participation rate enhances the reliability of the findings and supports contextual inference within the educational setting under study. Nevertheless, some cases of non-participation may have been attributable to practical factors such as student availability, willingness to participate, or the presence of additional disabilities accompanying hearing impairment. These considerations were taken into account when interpreting the findings; therefore, the results should be understood within the studied context rather than generalized to all DHH individuals at the national level.

In the qualitative phase, a purposive subsample of 25 HOH female students was selected from the original quantitative sample (*N* = 69) for in-depth individual interviews. This selection was not a random reduction but a deliberate process aligned with the explanatory mixed-methods design adopted in the study. Following the quantitative phase, participants were screened based on predefined criteria, including: (1) the relevance of participants’ characteristics to the study objectives, as all participants were enrolled in inclusive schools in Al-Ahsa Governorate; (2) variation in levels of psychological hardiness based on scores obtained in the quantitative phase; (3) the feasibility of effective communication during interviews, as HOH students were selected because communication with them was more manageable than with some students with profound deafness, particularly when discussing complex psychological issues; (4) willingness to participate in the individual interviews; and (5) the inclusion of female students only, due to logistical and cultural considerations associated with conducting the study in the Saudi educational context. Specifically, schools are gender-segregated, and access to female students requires adherence to institutional and cultural protocols that facilitate research within female educational settings. This made it more feasible for the researchers to conduct in-depth data collection in girls’ schools while ensuring compliance with ethical and administrative requirements. Accordingly, the findings should be interpreted within the scope of female DHH students, and future research is recommended to include male participants to enable comparative analysis and enhance the generalizability of the results. This approach ensured representation of diverse response patterns and enabled an in-depth exploration of the study phenomenon. Detailed demographic characteristics of the participants are presented in Tables 1, 2.

**TABLE 1:** Characteristics of the study sample who completed the scale.

Variable	Category	Frequency	Percentage
Degree of hearing loss	Deaf	17	24.6
	HOH	52	75.4
Grade level	Grade 7	13	18.8
	Grade 8	8	11.6
	Grade 9	14	20.3
	Grade 10	7	10.1
	Grade 11	12	17.4
	Grade 12	15	21.7
Type of hearing assistive device	Hearing aid	30	43.5
	Cochlear implant	17	24.6
	No assistive device	22	31.9
Parental hearing status	Both parents deaf/HOH	2	2.9
	Both parents hearing	61	88.4
	One parent deaf/HOH and the other hearing	6	8.7
Preferred communication mode	Oral communication	30	43.5
	Bilingual communication	21	30.4
	Sign language	18	26.1
Type of educational environment attended	Partial inclusion programs	6	8.7
	Full inclusion programs	42	60.9
	Al-Amal Institute in addition to partial inclusion programs	21	30.4

**TABLE 2:** Characteristics of the study sample who completed the individual interviews.

Variable	Category	Frequency	Percentage
Degree of hearing loss	Deaf	0	0
	HOH	25	100
Grade level	Grade 7	2	8
	Grade 8	5	20
	Grade 9	6	24
	Grade 10	3	12
	Grade 11	7	28
	Grade 12	2	8
Type of hearing assistive device	Hearing aid	12	48
	Cochlear implant	10	40
	No assistive device	3	12
Parental hearing status	Both parents deaf/HOH	6	24
	Both parents hearing	19	76
	One parent deaf/HOH and the other hearing	0	0
Preferred communication mode	Oral communication	18	72
	Bilingual communication	7	28
Type of educational environment attended	Partial inclusion programs	10	40
	Full inclusion programs	15	60
	Al-Amal Institute in addition to partial inclusion programs	0	0

### 3.3 Instruments

The present study employed the following instruments:

#### 3.3.1 Psychological hardiness scale (developed by the researchers)

The Psychological Hardiness Scale consisted of 24 items distributed across three main dimensions: commitment, control, and challenge. The development of the scale was informed by a review of the relevant literature and psychometrically validated measures in the field of psychological hardiness, particularly studies involving DHH individuals (Crowe, 2022; Gao et al., 2023; Mokhaimer, 2011; Xu et al., 2025). To ensure content validity, the initial version of the scale was reviewed by five experts in special education and psychology, who evaluated the clarity of the items and their appropriateness for the target population. Based on their feedback, several linguistic and wording revisions were made to improve item clarity and strengthen both face and content validity. To examine internal consistency, Pearson correlation coefficients were calculated between the items and the dimensions of the scale. The correlation coefficients ranged from 0.507 to 0.895, and all were statistically significant at *p* < 0.01, indicating an acceptable level of internal consistency. Reliability was further assessed using Cronbach’s alpha, which yielded a coefficient of 0.955, indicating a high level of reliability.

##### 3.3.1.1 Scale structure

Based on the validity and reliability procedures, the final version of the instrument consisted of two sections:

1. Section I: demographic variables, including degree of hearing loss, grade level, type of hearing assistive device used, preferred communication mode, parental hearing status, and type of educational environment attended.
2. Section II: the Psychological Hardiness Scale, comprising 24 items distributed across the three dimensions of commitment, control, and challenge.

Participants’ responses were rated on a four-point Likert scale:1 = never, 2 = rarely, 3 = often, 4 = always (see Supplementary Appendix A).

#### 3.3.2 Individual interviews

Semi-structured individual interviews were used as the primary tool for collecting qualitative data, as they provide the flexibility necessary to explore participants’ experiences and perceptions in depth while maintaining a systematic methodological framework (Abu’Allam, 2011). The interview guide included main and follow-up questions focusing on two key areas: factors influencing psychological hardiness among HOH female students, and strategies used to enhance psychological hardiness. The interviews were conducted with (25) HOH participants who had previously completed the Psychological Hardiness Scale in the quantitative phase (see Supplementary Appendix B). Each interview lasted approximately (50–60) minutes.

During the interviews, field notes were recorded to document contextual information, non-verbal cues, and initial analytical impressions. All interviews were audio-recorded with participants’ consent and subsequently transcribed verbatim for analysis. It should be noted that the qualitative interviews were conducted exclusively with hard-of-hearing (HOH) students rather than students with profound deafness. This selection was intentional, as HOH participants possess sufficient residual hearing to enable effective oral communication, and their speech production was generally clear and understandable. In contrast, students with profound deafness participated only in the quantitative phase (i.e., the scale) and were not included in the interview stage. The interviews were conducted face-to-face by the researchers themselves with the HOH students in the resource rooms within their respective schools. The interviews were not video-recorded due to the absence of parental consent, particularly considering cultural sensitivities and restrictions associated with female participants. Sign language was not used during the interviews, as all participants were able to communicate verbally without the need for interpretation. In this context, sign language served solely as a means of clarification during quantitative data collection, and participants’ responses were recorded in the standard format of the scale. Detailed note-taking during and immediately after the interviews further supported accurate data capture.

### 3.4 Data collection procedures

#### 3.4.1 Quantitative phase

After obtaining the necessary ethical and administrative approvals, the researchers visited the target schools, explained the objectives of the study to the participants, and obtained their consent to participate. The Psychological Hardiness Scale was then distributed in paper format to all (74) DHH female students enrolled in inclusive schools in Al-Ahsa. A total of (69) complete responses were obtained. Responses from the remaining students could not be collected due to absence, unwillingness to participate, or the presence of additional disabilities associated with hearing impairment.

#### 3.4.2 Qualitative phase

Following the analysis of the quantitative data, participants for the qualitative phase were selected using purposive sampling according to the criteria specified in the participant section. The qualitative phase involved several main steps: (1) Preparation phase: reviewing the interview guide and establishing its appropriateness through expert feedback from specialists in qualitative research; (2) Data collection phase: conducting the individual interviews; (3) Analysis phase: analyzing the qualitative data and identifying the main themes. During the interviews, communication procedures were adapted to suit participants’ needs. For example, the mode of communication was selected according to each participant’s preferences and their use of hearing assistive devices, and participants were allowed to choose the communication method most suitable for them. The interviews were conducted face-to-face in a quiet setting to ensure privacy, enhance participant comfort, and encourage open expression of views and experiences. Data collection continued until thematic saturation was reached, that is, until no new analytically meaningful themes emerged.

### 3.5 Data analysis

Quantitative data were analyzed using SPSS version 20. Frequencies, means, and standard deviations were calculated to determine the level of psychological hardiness among the participants. Qualitative data were analyzed using thematic analysis, which focuses on identifying underlying meanings in the data by examining patterns and relationships across themes (Al-Abdulkareem, 2012). Thematic analysis is one of the most widely used approaches in qualitative research because it relies on systematic coding of textual data and field notes and contributes to a deeper theoretical understanding of the phenomenon under investigation (Robson and McCartan, 2016). The six phases of thematic analysis proposed by (Braun and Clarke, 2006) were followed: (1) familiarization with the data, (2) generating initial codes, (3) searching for themes, (4) reviewing themes, (5) defining and naming themes, and (6) producing the final report.

### 3.6 Trustworthiness and credibility of the qualitative data

To ensure the credibility and trustworthiness of the qualitative findings, several methodological procedures recommended in the relevant literature were followed (Abu’Allam, 2011; Braun and Clarke, 2006). First, methodological triangulation was employed by integrating two sources of data—the Psychological Hardiness Scale and the individual interviews—which helped reduce bias and strengthen the validity of the findings. Second, expert review was conducted. Four faculty members specializing in education reviewed the interview questions, and revisions were made based on their feedback, including deleting some questions, rewording others, and adding more in-depth questions. Third, systematic coding was applied during data analysis. The researchers maintained analytic memos to document emerging ideas and patterns throughout the analysis process. Finally, member checking was conducted by providing participants with transcripts of their interviews after transcription and asking them to review them to ensure accuracy and faithful representation of their experiences.

## 4 Results

### 4.1 Findings related to the first research question

To answer the first research question, means and standard deviations were calculated for the participants’ responses regarding the level of psychological hardiness among DHH female students in the Kingdom of Saudi Arabia. The results are presented in Table 3.

**TABLE 3:** Means and standard deviations of participants’ responses regarding the level of psychological hardiness.

No.	Item	Mean	SD	Level of agreement	Rank
1	No matter what difficulties I face, I am able to achieve my goals.	2.54	1.051	Moderate	20
2	I believe that I am no less than others because of my hearing loss.	2.65	1.055	Moderate	17
3	I have principles that I adhere to and maintain.	3.84	0.407	High	1
4	I have goals that I strive to achieve.	3.29	0.925	High	5
5	I remain committed to participating in social or school activities despite my hearing loss.	2.91	0.966	Moderate	11
6	I remain committed to my work even when it causes me stress.	2.57	1.206	Moderate	18
7	I believe that stress and difficulties are part of human life.	2.94	1.042	Moderate	10
8	I am confident in my ability to succeed in life despite hearing loss.	3.23	1.017	High	6
9	I feel responsible toward others and take the initiative to help them.	3.54	0.739	High	2
10	I make my own decisions and do not rely on someone else to make them for me.	2.96	1.021	Moderate	9
11	When I make future plans, I am confident in my ability to carry them out.	3.03	0.939	High	7
12	My success in life depends on my effort rather than luck.	3.32	0.813	High	3
13	I do not allow negative situations caused by hearing loss to affect my life.	2.04	1.143	Moderate	24
14	I plan my life and do not leave it to external circumstances.	2.83	1.111	Moderate	12
15	I believe that work and effort play an important role in my life.	3.32	0.883	High	3
16	I believe that change is a part of life, and what matters is the ability to deal with it successfully.	2.99	0.978	Moderate	8
17	I believe that the enjoyment of life lies in one’s ability to face its challenges.	2.68	1.105	Moderate	16
18	When I face a problem because of my hearing loss, I look for solutions instead of giving up.	2.52	1.079	Moderate	21
19	I am curious and eager to explore new things.	2.72	1.110	Moderate	15
20	I find satisfaction in facing challenges and difficulties and working to solve them.	2.42	1.006	Moderate	23
21	I keep trying until I overcome any problem I face.	2.75	0.976	Moderate	13
22	I am prepared for possible changes in my life.	2.75	1.117	Moderate	13
23	After solving one problem, I feel motivated to move on to solving another.	2.51	0.980	Moderate	22
24	I am not afraid to try new things because of my hearing loss.	2.57	1.254	Moderate	18
Overall mean	2.87	0.634		

The results in the previous table indicate that the level of psychological hardiness among DHH female students in Saudi Arabia was at a moderate level, with an overall mean of 2.87 and a standard deviation of 0.634, which falls within the moderate category according to the adopted rating scale. This finding suggests that the students possess an acceptable level of psychological hardiness, although it does not reach a high level, indicating room for further enhancement through counseling programs or appropriate psychological and educational interventions.

At the item level, the findings showed variation in response means. Some items received relatively high mean scores, most notably “I have principles that I adhere to and maintain” (*M* = 3.84), followed by “I feel responsible toward others and take the initiative to help them” (*M* = 3.54). These findings suggest that the students demonstrate a relatively strong sense of personal values, responsibility, and self-reliance, which are core components of psychological hardiness.

By contrast, some items showed relatively lower mean scores, particularly those associated with coping with stress and challenges, such as “I do not allow negative situations caused by hearing loss to affect my life” (*M* = 2.04) and “I find satisfaction in facing challenges and difficulties and working to solve them” (*M* = 2.42). These findings indicate that the students may experience difficulties in dealing with stress and negative situations related to hearing loss, which may in turn affect their capacity to confront challenges effectively.

A rating scale was adopted to interpret participants’ mean scores on the scale items in order to classify levels of psychological hardiness as low, moderate, or high, as shown in Table 4.

**TABLE 4:** Mean score ranges and corresponding levels.

Mean score	Level
1 to less than 2	Low
2 to less than 3	Moderate
3 to 4	High

### 4.2 Findings related to the second research question

To answer the third research question, the Conceptual Analysis Method (CAM) was used to analyze data from the semi-structured interviews with the participants. The analysis yielded four major themes influencing psychological hardiness among HOH female students, as follows:

#### 4.2.1 Family factors

The interview findings indicated that the family constitutes one of the most important sources influencing the psychological hardiness of HOH female students, either positively or negatively, depending on the nature of the support and family interactions they receive. For example, one participant referred to the effect of social comparison within the family and the presence of deaf or HOH family members on her self-perception, stating: “I do not wear a hearing aid because no one in my family wears one, so I feel that I look wrong when I wear it” (P22). Another participant pointed to the negative impact of family-based stigma on her emotional wellbeing, saying: “I feel very upset when I hear someone in my family talking about my disability and my inability to hear, as if I am somehow lacking compared with others. I may accept it from people, but I cannot accept it from my family” (P16). In the context of family support in adapting to hearing impairment, one participant said: “I have a deformity in the outer ear. When I was young, my mother used to cover my ear with my hair so that people would not talk about me. But when I grew up, I got used to it and started saying that this is how God created me and I have to accept it” (P3). These findings suggest that family support and emotional acceptance can significantly contribute to strengthening psychological hardiness, whereas lack of understanding or social sensitivity within the family may increase feelings of difference or isolation.

#### 4.2.2 Societal factors

The interviews showed that societal attitudes toward hearing loss constitute an important factor affecting the psychological hardiness of HOH female students. Many participants reported experiencing negative comments or stigma in public spaces. For example, one participant stated: *“People keep bullying me everywhere, even when I am with my family or in public places, so I no longer like leaving the house or attending social gatherings, and I do not like wearing my hearing aid because then people will know I am HOH”* (P18). Another participant emphasized that the level of public awareness plays an important role in their psychological experiences, saying: *“The level of awareness in society about deafness or hearing loss affects my strength and mental state a lot. I often hear people saying, ‘Poor thing, she is still young, why does she wear a hearing aid?”’* (P12). Another participant described her experience of bullying associated with additional health-related issues: *“I was constantly bullied. They used to say that I was cross-eyed because there is a problem with the lens in my eye. I gave up the hearing aid so that they would not bully me even more. I do not care if my hearing gets better; what matters is that I feel relieved from people’s words”* (P20). Similarly, another participant said: *“I do not have an outer ear, so I undergo surgeries continuously. They take part of my body to reconstruct the ear, People often ask me what is wrong with your ear, it has been cut?, and this affects my mental state all the time”* (P17). One participant also described the social impact of speech difficulties, stating: *“I have a speech problem, so when I speak, they laugh at me. It hurts me deeply when I see them laughing; it feels like something is squeezing my heart”* (P11). These findings suggest that limited societal awareness and stigma associated with hearing impairment may constitute major barriers to the development of psychological hardiness among these students.

#### 4.2.3 Peers

The individual interviews revealed that peer relationships are among the most influential factors in the psychological hardiness of HOH female students. Many participants reported difficulties in forming friendships, as well as experiences of bullying or social exclusion, which affected their sense of social acceptance and self-confidence. For example, one participant expressed her sense of social isolation by saying: *“I always cry because I do not have friends, so I decided to turn to online friendships because they do not know that I am HOH”* (P4). Another participant noted the lack of support from hearing students at school: *“The hearing girls do not help HOH girls. They always laugh at us and raise their voices, and when we ask a question, no one responds”* (P6). In the context of bullying related to physical appearance, one participant said: *“My heart hurts when they bully my appearance, and this is the reason why I have very few friendships because of the problems I face with them”* (P11). Another participant described her experience in a mainstream school by saying: *“I was in a regular school. I was the only one who was HOH, and even my only friend used to tell me to hide the hearing aid under my hair so that no one would see it because she felt embarrassed walking with me”* (P22). Experiences of bullying also appeared to begin in the early stages of schooling, as one participant stated: *“Once in kindergarten, a girl said to me, “What happened to your ear? Who cut it?’ I started hating going to kindergarten because of the bullying I heard from my classmates.”*

It was also observed during the group session held before the individual interviews, which aimed to gain a general understanding of the students, that there were some tensions in the relationships among the students themselves. For instance, one participant refused to hold her classmate’s hand during one of the group activities because of a previous disagreement between them. The interviews also revealed a clear degree of reservation among some participants when discussing personally influential experiences, reflecting the sensitivity of such topics for them. These findings indicate that the quality of peer relationships plays a pivotal role in either strengthening or weakening psychological hardiness, as positive social support may promote psychological adjustment, whereas bullying or social isolation may intensify psychological distress.

#### 4.2.4 Teachers

The qualitative findings indicated that teachers represent an influential factor in the psychological hardiness of HOH female students, as they may serve either as a source of psychological support or as a source of stress and negative experiences. For example, one participant referred to some negative experiences with teachers, saying: “Teachers sometimes laugh at us, and this affects me a lot, even more than peers. They are supposed to be role models and help us, not laugh at us” (P13). Another participant described a particularly difficult experience with mainstream education teachers, stating: “The general education teacher always underestimates our abilities. She says, “This is an inclusion student, of course there will be no creativity, so why should I waste my effort on her?”” (P19). By contrast, some participants referred to the positive influence of teachers on their emotional wellbeing. One participant said: “There are teachers who always help us and defend us in the classroom, and this has a strong effect on our wellbeing” (P2). These findings indicate that supportive teaching practices can play an important role in enhancing psychological hardiness, whereas unsupportive practices within the school environment may increase psychological stress among students. Taken together, these themes indicate that psychological hardiness is not shaped solely by individual factors, but is also influenced by social experiences and daily interactions within educational, familial, and societal contexts.

### 4.3 Findings related to the third research question

Analysis of the interviews using the Conceptual Analysis Method (CAM) yielded a set of strategies used by HOH female students to strengthen their psychological hardiness and adapt to the social and psychological challenges they face in everyday life. These strategies were classified into five major themes, as follows:

#### 4.3.1 Faith in God and reliance on Him

Many participants indicated that faith in God and reliance on Him constitute a core source of psychological strength and inner peace. The spiritual dimension appeared to help them cope with the pressures and challenges associated with hearing loss. Participants explained that turning to faith, remembrance of God, and religious meanings gave them a sense of psychological comfort and strengthened their ability to face difficult situations with patience and confidence. For example, one participant stated: “Whenever I feel anxious, I remember the verse: “Do not fear; indeed, I am with you both; I hear and I see”” (P2). Another participant expressed her view of life from a faith-based perspective that strengthened her ability to accept challenges and deal with them positively: “It is natural for us to have problems. Life means that I experience emotions in every situation I go through and face it with faith and strength” (P22). These statements show that faith was not merely a source of psychological comfort, but also served as a cognitive framework through which participants interpreted life difficulties and approached them with greater acceptance and flexibility. Thus, faith in God and reliance on Him may be considered an important psychological resource supporting psychological hardiness among DHH female students by enhancing a sense of meaning and the capacity to adapt to difficult circumstances.

#### 4.3.2 Social support and relationship improvement

The qualitative findings showed that social support is one of the most important factors contributing to the enhancement of psychological hardiness among HOH female students. Participants emphasized that having trusted people with whom they could speak openly helped reduce psychological stress and gave them a sense of safety and belonging. Many students indicated that family relationships, particularly with parents and sisters, played a central role in providing emotional support and encouraging them to face challenges associated with hearing loss. For example, one participant stated: “I like talking to my sister because I tell her everything that happens to me, and she does not tell anyone else” (P24), which reflects the importance of a trusting and confidential relationship that allows the student to express her feelings and experiences without fear of judgment. Another participant highlighted the supportive role of her father in strengthening her self-confidence and motivating her to pursue her aspirations, saying: “My relationship with my father is very strong, almost as if he is more than just a father. He likes to encourage me and say things that make me stronger. He tells me: “You can do it, I will support you, enter the specialization you want, you are capable of it.”” In addition, some participants mentioned that friends represented an important source of psychological and social support by providing an environment of understanding and emotional sharing. Others referred to the importance of support received from family members such as mothers and aunts. One participant summarized the meaning of social support by saying: “All we ask is that people look at us like everyone else and do not diminish our worth” (P1). This statement reflects the students’ need for social recognition and acceptance, both of which contribute to a sense of belonging and human dignity and positively influence their psychological hardiness.

#### 4.3.3 Coping and stress-management strategies

The interview findings showed that HOH female students use a variety of psychological coping strategies to deal with daily stress and difficult situations associated with their lived experiences. These strategies included emotion-regulation approaches, reflective thinking, and distraction techniques to reduce tension. One participant referred to temporary withdrawal as a means of dealing with stressful situations, saying: “I always face my problems with silence… I go to my room and distance myself from problems” (P1). Another participant described relying on reflective thinking and re-evaluation of situations after they occurred, stating: “I started using the “what if” strategy… when I get angry about something, I later blame myself and say, “I wish I had said this instead”” (P11). some participants also indicated using distraction strategies to reduce tension and negative emotions. One participant said: “I think about something else that makes me forget the issue, such as cooking, cleaning, or going to the market” (P5). This behavior reflects an attempt to redirect attention toward other activities that help reduce persistent rumination about the problem, thereby alleviating psychological stress.

#### 4.3.4 Developing self-confidence and emotional balance

Participants explained that strengthening self-confidence is one of the important strategies that helps them face negative situations and deal with difficult social experiences such as bullying or being underestimated because of hearing impairment. One participant noted that she tries to deal with such situations by strengthening her inner psychological stance, saying: *“When I am exposed to a situation such as bullying, I say to the person: “As you do, so shall it be done to you””* (P12). Some participants also expressed adopting a positive perspective toward their hearing impairment as part of their identity rather than as an absolute barrier. One participant stated: *“For me, being unable to hear is better than being unable to see or walk… at least I use sign language and lip reading, and people can understand me, so my problem can be solved”* (P23). This reflects the adoption of a positive interpretive framework that reduces the psychological burden of disability and promotes self-acceptance.

#### 4.3.5 Self-change and personal development

The interviews further showed that some students possess a strong motivation for self-development and for improving aspects of their personalities in order to strengthen their ability to cope with social and psychological challenges. One participant expressed her desire to change certain personal characteristics that she believed made her vulnerable to unfair treatment, stating: *“I wish I could change my excessive kindness. I see people always treating me badly, and I stay silent”* (P2). Another participant expressed a desire to improve her emotional regulation skills, saying: *“Even when someone talks to me in a loud voice, I feel as if they are shouting at me and I start crying. My excessive sensitivity has exhausted me, and I wish I could get rid of it”* (P7). These statements indicate that some participants were aware of the emotional challenges they faced and were motivated to develop better emotion-regulation and coping skills for stressful situations.

Overall, these findings suggest that HOH female students rely on a diverse set of psychological and social resources and strategies to strengthen their capacity to cope with everyday challenges. Collectively, these factors support psychological hardiness and enable the students to deal with hearing loss–related stressors with greater resilience and adjustment.

### 4.4 Findings related to the forth research question

To answer the mixed-methods research question, the findings from the quantitative and qualitative phases were integrated in order to interpret the level of psychological hardiness among DHH female students in the Kingdom of Saudi Arabia in light of the influencing factors and supportive strategies identified through the interviews. The quantitative findings showed that the level of psychological hardiness was moderate, with an overall mean of 2.87, indicating that the students possessed an acceptable level of psychological hardiness, although this level still requires further strengthening. The qualitative findings helped explain this moderate level by showing that psychological hardiness among the students is shaped within a complex context in which social, familial, and educational experiences interact with psychological, personal, and spiritual resources. These factors may influence psychological hardiness either positively or negatively.

The integrated findings indicate that stressors such as difficulties in peer relationships, exposure to bullying, limited societal awareness, and certain unsupportive practices from family members or teachers contributed to weakening some aspects of psychological hardiness, particularly those related to confronting negative situations, persisting in dealing with stress, and finding meaning or satisfaction in challenges. This is evident in the relatively lower means of certain quantitative items, such as *“I do not allow negative situations caused by hearing loss to affect my life”* and *“I find satisfaction in facing challenges and difficulties and working to solve them.”* This pattern is consistent with the interview findings, which showed that many students experience repeated incidents of bullying, exclusion, or embarrassment related to hearing loss or the use of hearing assistive devices, thereby making their ability to resist the psychological impact of negative experiences less stable.

In contrast, the qualitative findings showed that there are also supportive factors that help maintain an acceptable level of psychological hardiness despite these challenges. Most notably, these include faith in God, social support from certain family members or friends, the development of self-confidence, and the use of psychological coping strategies for managing stress. These qualitative findings help explain the higher means observed for items related to personal values, responsibility, and the belief in the importance of effort in achieving success, such as *“I have principles that I adhere to and maintain,”* and *“I feel responsible toward others and take the initiative to help them.”* This suggests that, despite the pressures they face, the students do not lack a values-based foundation or personal motivation; rather, they need more supportive environments in order for these internal resources to develop into stronger and more stable psychological hardiness.

The integrated findings also suggest that supportive strategies do not operate independently of influencing factors; rather, they play both an interpretive and a compensatory role. When a student encounters bullying or insufficient support, turning to faith in God, or to a sister, father, or friend, or using distraction and emotion-regulation strategies, may help contain the negative psychological impact and prevent further deterioration in psychological hardiness. However, reliance by some students on strategies such as silence, withdrawal, or emotional suppression may provide temporary relief, but does not address the source of stress in a deeper way. This may help explain why psychological hardiness remained at a moderate, rather than a high, level.

Overall, the mixed-methods findings indicate that the moderate level of psychological hardiness among DHH female students is the result of a balance between two opposing forces. On the one hand, there are stressful factors, including bullying, isolation, social stigma, and insufficient support in some family and educational contexts. On the other hand, there are supportive resources and strategies, including faith in God, social support, self-confidence, and gradual adaptation to challenges. Accordingly, psychological hardiness did not decline to a low level because of the presence of these supportive resources; however, it also did not rise to a high level because of the continued influence of contextual and social pressures.

This finding suggests that strengthening psychological hardiness among DHH female students should not be limited to the development of individual skills alone, but should also involve improving the social, familial, and educational environment, raising public awareness, reducing bullying, and providing more inclusive and supportive educational practices, in addition to developing counseling programs that build on the sources of strength identified in the qualitative findings.

## 5 Discussion

The findings of the present study showed that the level of psychological hardiness among DHH female students was moderate. This finding is consistent with the results reported by Xu et al. (2025), who found that DHH students demonstrate moderate levels of psychological resilience. It is also in line with several studies indicating that hearing impairment may be associated with a range of psychological and social challenges, such as communication difficulties, social isolation, and stress related to interaction within predominantly hearing environments, all of which may be reflected in the level of psychological hardiness among this population (Eschenbeck et al., 2017; Kobosko et al., 2015; Mohamed, 2019; Mousa and Khalaf, 2017). These findings suggest that DHH individuals may possess psychological resources that help them adapt to stress; however, such resources may not always be sufficient to achieve high levels of psychological hardiness. This highlights the need for educational and counseling interventions that strengthen psychological immunity and develop effective coping skills (Faraj, 2025).

In this context, it is noteworthy that although many DHH individuals are exposed to experiences of social bias or marginalization (Crowe, 2019; Johnson et al., 2018; Mousley and Chaudoir, 2018), the findings of the current study suggest that they do not necessarily exhibit sharply reduced levels of psychological hardiness. This may be interpreted in light of the social and legislative developments that have taken place in recent years regarding the rights of persons with disabilities, which have improved access to education, services, and social support (Wardle, 2017). In addition, the expansion of social media use, the availability of captioning services, and access to digital education may have provided DHH individuals with greater opportunities for communication, learning, and the acquisition of adaptive coping strategies that help them deal more effectively with life challenges (Kimball et al., 2016; Wardle, 2017).

On the other hand, the qualitative findings revealed a set of psychological and social factors that influence psychological hardiness among HOH female students. This finding is consistent with several studies showing that the quality of family relationships and social support from the broader community are important factors in enhancing hardiness and resilience among DHH individuals (Crowe, 2019; Johnson et al., 2018). Similarly, (Xu et al., 2025) found that individuals with higher levels of social support or broader peer networks tend to exhibit higher levels of resilience. Social support also appears to reduce negative school experiences among deaf children and to promote their psychological and social adjustment (Fellinger et al., 2009). Moreover, some studies suggest that a supportive social environment and a sense of belonging to the Deaf community may function as protective factors against psychological stress and reduce the likelihood of stress-related mental health difficulties (Lambez et al., 2020).

With regard to the family context, the present findings are consistent with Mousley and Chaudoir (2018), who noted that many deaf individuals grow up in families whose members do not possess sign language skills, which may limit effective family communication and contribute to negative emotions such as frustration or anger. The effects of such communication barriers may extend into later life stages, potentially affecting the formation of positive identity and the enhancement of psychological wellbeing among deaf individuals.

In addition, the findings of the present study point to the positive role of supportive strategies in enhancing psychological hardiness among HOH female students. The literature suggests that psychological hardiness is not a fixed trait, but rather a dynamic psychological construct that can be developed through educational experiences and psychological and social support. Some studies have shown that targeted psychological interventions can improve psychological quality of life, strengthen psychological reassurance, and foster resilience among DHH individuals (Al-Kafouri et al., 2022; Faisal, 2013; Ibrahim and Yousif, 2015). Other studies have shown that such strategies may contribute to improving social skills, reducing loneliness, and alleviating some stress-related psychological difficulties (Ahmed et al., 2021; Khairallah et al., 2011).

The literature also indicates that hardiness and resilience are associated with several positive indicators of psychological adjustment, such as psychological security, positive affect, and academic and emotional adjustment (Badr Al-Din et al., 2022; Hassan and Ammar, 2020; Mohamed et al., 2022). In this regard, strengthening psychological hardiness may enhance the ability of HOH individuals to cope with challenges associated with hearing impairment, particularly given that communication difficulties may reduce access to social support networks and increase the likelihood of social withdrawal (Ogawa et al., 2019).

Accordingly, the findings of the current study suggest that fostering psychological hardiness extends beyond merely reducing psychological difficulties; it also contributes to the development of positive psychological resources that help HOH female students adapt more effectively to hearing loss–related challenges and achieve better levels of psychological and social adjustment.

### 5.1 Conclusion

The present study concluded that the level of psychological hardiness among DHH female students in Saudi Arabia was moderate, indicating that they possess an acceptable capacity to cope with challenges, although this capacity still requires further strengthening and support. The qualitative findings revealed that psychological hardiness is shaped by several contextual factors, most notably peers, family, society, and teachers, all of which may either support or undermine hardiness depending on the nature of the interactions and the support available. The study also showed that students rely on a number of strategies to strengthen their psychological hardiness, most notably faith in God and reliance on Him, social support, coping and stress-management strategies, self-confidence and emotional regulation, and self-development. These findings underscore the importance of adopting counseling and educational programs that enhance psychological hardiness among DHH female students, alongside raising public awareness and providing more inclusive and supportive educational environments that respond to their psychological and social needs.

### 5.2 Recommendations and future research

In light of the findings of the study, there is a need to adopt comprehensive educational and psychological approaches that enhance psychological hardiness among DHH female students within a supportive environment that takes into account the particularity of their psychological and social needs. The findings indicate the importance of developing specialized counseling and psychological programs within educational institutions that target the development of stress-coping skills, self-confidence, and the ability to confront challenges among this population. It is also important to empower teachers and educational practitioners through specialized training programs that focus on psychologically supportive teaching practices and effective educational strategies for working with DHH female students, thereby contributing to more inclusive and equitable educational environments. Raising public awareness of hearing impairment is another essential step in reducing the social stigma and bullying that these students may face, which calls for awareness campaigns and community initiatives that foster a culture of acceptance of difference and support the inclusion of individuals with hearing impairment in society. The findings also emphasize the importance of the family as a primary source of psychological and social support, which highlights the need to design guidance programs for parents that enhance their understanding of their daughters’ needs and equip them with the skills necessary to provide appropriate emotional and educational support. Furthermore, the findings point to the importance of supporting appropriate communication modes, such as sign language and bilingual communication, within educational environments in order to reduce the pressures associated with communication difficulties and strengthen students’ sense of competence and belonging.

At the level of future research, there is a need for more in-depth studies that explore the psychological and social factors associated with psychological hardiness among DHH individuals in different educational and cultural contexts. Experimental studies examining the effectiveness of counseling or training programs designed to enhance psychological hardiness could also provide applied evidence to inform educational practices. Comparative studies between DHH students and hearing students are also recommended in order to better understand differences in patterns of psychological adjustment and the factors influencing them. In addition, future research may explore the relationship between psychological hardiness and other psychological variables such as resilience, self-esteem, and psychological wellbeing. Longitudinal studies may also contribute to tracking the development of psychological hardiness among DHH students across different educational stages, thereby providing deeper insight into their mechanisms of psychological adaptation and the factors that enhance their long-term capacity to face challenges.

### 5.3 Limitations

Despite the importance of the findings of the present study, they should be interpreted in light of several methodological and contextual limitations. First, the study relied on a relatively small and context-specific qualitative sample that was purposively selected in order to deepen the interpretive understanding of the phenomenon under investigation rather than to achieve statistical representativeness of the broader population. Accordingly, the findings are not intended for probabilistic generalization; rather, they provide analytical and contextual insights into the level of psychological hardiness and the factors influencing it within a specific social, cultural, and educational context. Second, the study adopted a sequential explanatory mixed-methods design with a notable emphasis on the qualitative component, which enabled a deeper understanding of participants’ experiences, perceptions, and coping mechanisms. However, this methodological design focuses more on interpretation and exploration than on testing causal or predictive relationships between variables. Therefore, the findings should be understood as a contribution to the theoretical and contextual understanding of the phenomenon rather than as evidence of direct causal relationships or universally generalizable psychological patterns. Third, the current study focused on psychological hardiness as one indicator of positive mental health among DHH female students, without extending the analysis to other related psychological variables such as resilience, quality of life, or psychological and social support. Including such variables in future research may provide a more comprehensive understanding of the psychological and emotional dimensions associated with the experiences of students with hearing impairment.

In addition, limited variability was observed in responses to some scale items. This low variability may reflect a degree of homogeneity within the study sample, as participants shared similar contextual characteristics (i.e., female DHH students enrolled in inclusive schools within the same geographic region). It may also be related to the nature of the construct being measured—psychological hardiness—which could lead to more consistent response patterns among individuals exposed to comparable educational and social environments. Furthermore, the possibility of socially desirable responding cannot be entirely ruled out, particularly given the school-based data collection context. To mitigate this effect, participants were assured of anonymity and confidentiality, and the purpose of the study was clearly explained to encourage honest responses. Nevertheless, this limited variability was taken into consideration during data interpretation, and the findings are therefore discussed within the specific context of the study rather than generalized beyond it.

Finally, the study relied heavily on participants’ self-reported narratives, which may have been influenced by multiple factors, including preferred communication styles, cultural norms related to emotional expression, and the degree of comfort participants felt in disclosing their personal experiences. However, this should not necessarily be viewed as a methodological weakness; rather, it represents a core feature of qualitative research, which privileges the subjective meaning of human experience as a legitimate source of scientific knowledge. Accordingly, future studies may build on these findings by broadening the scope of research to include different institutional and educational contexts, adopting longitudinal designs to examine the development of psychological hardiness and coping mechanisms over time, and conducting comparative studies across different educational and cultural settings to gain a deeper understanding of how experiences and coping processes are shaped among this population.

## Data Availability

The original contributions presented in the study are included in this article/Supplementary material, further inquiries can be directed to the corresponding author.
