# Supplementary material for: Psychological hardiness among deaf and hard-of-hearing female students in Saudi Arabia: a mixed-methods analysis of influencing factors and enhancement strategies
Source: Front Psychol. 2026 May 26;17:1831967. doi: 10.3389/fpsyg.2026.1831967 (PMC13246376; doi:10.3389/fpsyg.2026.1831967)
Supplement: Supplementary file 1 [file Supplementary_file_1.docx]

**Appendix A**

**Dear Student,**

The researchers are conducting a scientific study entitled: “The Effectiveness of an Integrative Therapy–Based Program in Improving Psychological Hardiness among Hard-of-Hearing Female Students in Al-Ahsa Governorate.” This scale will be administered to a group of hard-of-hearing female students in Al-Ahsa Governorate. By completing this questionnaire, you will help the researchers better understand this topic and grant permission to include your responses in the study. The results will be used to propose improvements in counseling services and educational curricula at the intermediate level and beyond.

The scale presented to you includes a number of statements describing personal characteristics that may or may not apply to you. Each statement is followed by several response options. Please read each statement carefully and select the option that best reflects your opinion.

Please note that there are no right or wrong answers, and your responses will remain confidential and will be used for scientific research purposes only. The research instrument has received ethical approval from the Research Ethics Committee at King Faisal University (Approval No. Ethics3681), dated 21 October 2025. Your participation in this study is voluntary. Your identity will not be disclosed in the questionnaire. You may stop completing the questionnaire at any time without any penalty. There are no risks associated with completing this questionnaire.

With sincere appreciation,

**The Researchers:**

Dr. Reem Abdullatif Al-Arfaj

Dr. Nourah Ibrahim Al-Bash

**Email**:
raalarfaj@kfu.edu.sa
nalbash@kfu.edu.sa

**Mobile:**
+96656066999
+966547344111

**Student Demographic Information**

**Grade Level**

- First Intermediate
- Second Intermediate
- Third Intermediate
- First Secondary
- Second Secondary
- Third Secondary

**Type of Hearing Assistive Device**

- Hearing aid
- Cochlear implant
- No assistive device

**Parents’ Hearing Status**

- Both parents deaf / hard of hearing
- Both parents hearing
- One parent deaf / hard of hearing and the other hearing

**Communication Method Used**

- Sign language
- Verbal communication
- Bilingual communication

**Type of Educational Environment Attended Across All School Levels**

- Al-Amal Institute
- Partial inclusion programs
- Full inclusion programs
- Al-Amal Institute plus partial inclusion programs
- Al-Amal Institute plus full inclusion programs

**Degree of Hearing Loss**

- Deaf
- Hard of hearing

**Scale Items**

| **No.** | **Statement** | **Always** | **Often** | **Rarely** | **Never** |
| --- | --- | --- | --- | --- | --- |
| 1 | Regardless of the difficulties I face, I can achieve my goals. |  |  |  |  |
| 2 | I believe that I am not inferior to others because of my hearing loss. |  |  |  |  |
| 3 | I have principles that I adhere to and maintain. |  |  |  |  |
| 4 | I have goals that I strive to achieve. |  |  |  |  |
| 5 | I commit to participating in social or school activities despite my hearing loss. |  |  |  |  |
| 6 | I remain committed to my work regardless of the pressure it causes me. |  |  |  |  |
| 7 | I believe that stress and difficulties are part of human life. |  |  |  |  |
| 8 | I trust my ability to succeed in life despite my hearing loss. |  |  |  |  |
| 9 | I feel responsible toward others and take the initiative to help them. |  |  |  |  |
| 10 | I make my own decisions and do not rely on others to make them for me. |  |  |  |  |
| 11 | When I set future plans, I am confident in my ability to implement them. |  |  |  |  |
| 12 | My success in life depends on my effort rather than luck. |  |  |  |  |
| 13 | I do not allow negative situations caused by hearing loss to affect my life. |  |  |  |  |
| 14 | I plan my life and do not leave it to external circumstances. |  |  |  |  |
| 15 | I believe that work and effort play an important role in my life. |  |  |  |  |
| 16 | I believe that change is a part of life, and what matters is the ability to deal with it successfully. |  |  |  |  |
| 17 | I believe that the joy of life lies in the ability to face its challenges. |  |  |  |  |
| 18 | When I face a problem due to my hearing loss, I look for solutions instead of giving up. |  |  |  |  |
| 19 | I have curiosity and a desire to explore new things. |  |  |  |  |
| 20 | I enjoy facing challenges and difficulties and working to solve them. |  |  |  |  |
| 21 | I continue trying until I solve any problem I face. |  |  |  |  |
| 22 | I am prepared for the changes that may occur in my life. |  |  |  |  |
| 23 | After solving a problem, I feel motivated to move on and solve another. |  |  |  |  |
| 24 | I am not afraid to try new things because of my hearing loss. |  |  |  |  |

**Thank you**
