# Supplementary material for: Psychological hardiness among deaf and hard-of-hearing female students in Saudi Arabia: a mixed-methods analysis of influencing factors and enhancement strategies
Source: Front Psychol. 2026 May 26;17:1831967. doi: 10.3389/fpsyg.2026.1831967 (PMC13246376; doi:10.3389/fpsyg.2026.1831967)
Supplement: Supplementary file 2 [file Supplementary_file_2.docx]

**Appendix B**

**Individual Interview Guide**

Thank you for agreeing to participate in this interview. The interview aimed to explore the experiences of deaf and hard-of-hearing female students and identify the factors influencing their psychological hardiness and the strategies they use to cope with daily challenges.

1. Personal Experiences

- Can you describe your experience living with hearing loss in your daily life?
- What are the main challenges you face at school or in society because of hearing loss?

2. Family Support

- How does your family deal with your hearing loss?
- In what ways does your family support you when you face difficulties?

3. Social and Peer Relationships

- How would you describe your relationships with classmates and friends?
- Have you ever experienced exclusion or bullying due to hearing loss?

4. School Environment

- How do teachers usually interact with deaf or hard-of-hearing students?
- Have teachers helped you overcome academic or social challenges?

5. Coping Strategies and Psychological Strength

- When you face stressful situations or problems, how do you usually deal with them?
- What things help you feel stronger or more confident in difficult situations?

6. Closing Question

- Is there anything else you would like to share about your experiences as a deaf or hard-of-hearing student?

**Thank you**
